# Prevention Strategies and Early Diagnosis of Cervical Cancer: Current State and Prospects

**DOI:** 10.3390/diagnostics13040610

**Published:** 2023-02-07

**Authors:** Viktor V. Kakotkin, Ekaterina V. Semina, Tatiana G. Zadorkina, Mikhail A. Agapov

**Affiliations:** 1 Scientific and Educational Cluster MEDBIO, Immanuel Kant Baltic Federal University, A. Nevskogo St., 14, 236041 Kaliningrad, Russia; 2 Kaliningrad Regional Centre for Specialised Medical Care, Barnaulskaia Street, 6, 236006 Kaliningrad, Russia

**Keywords:** cervical cancer, prevention strategies, diagnostics, precision medicine, artificial intelligence, microbiome

## Abstract

Cervical cancer ranks third among all new cancer cases and causes of cancer deaths in females. The paper provides an overview of cervical cancer prevention strategies employed in different regions, with incidence and mortality rates ranging from high to low. It assesses the effectiveness of approaches proposed by national healthcare systems by analysing data published in the National Library of Medicine (Pubmed) since 2018 featuring the following keywords: “cervical cancer prevention”, “cervical cancer screening”, “barriers to cervical cancer prevention”, “premalignant cervical lesions” and “current strategies”. WHO’s 90-70-90 global strategy for cervical cancer prevention and early screening has proven effective in different countries in both mathematical models and clinical practice. The data analysis carried out within this study identified promising approaches to cervical cancer screening and prevention, which can further enhance the effectiveness of the existing WHO strategy and national healthcare systems. One such approach is the application of AI technologies for detecting precancerous cervical lesions and choosing treatment strategies. As such studies show, the use of AI can not only increase detection accuracy but also ease the burden on primary care.

## 1. Introduction

It was in 1996 that the World Health Association, the European Research Organization on Genital Infection and Neoplasia and the National Institutes of Health Consensus Conference on Cervical Cancer recognised the role of human papillomavirus (HPV) in cervical cancer development [1]. According to the degree of association with invasive tumours, HPV genotypes have been subdivided into those posing high oncogenic risk, low oncogenic risk and undetermined risk. High oncogenic risk (16, 18, 31, 33, 35, 39, 45, 51, 52, 56, 58, 59, 66, 68) is related to an increased risk of developing cervical cancer [2]. Low oncogenic risk (6, 11, 40, 42, 43, 44, 54, 61, 70, 72, 81, 89) is associated, in most cases, with no disease or benign epithelial lesions, such as anogenital and oropharyngeal warts [3]. Finally, undetermined risk HPVs (3, 7, 10, 27, 28, 29, 30, 32, 34, 55, 57, 62, 67, 69, 71, 74, 77, 83, 84, 85, 86, 87, 90, 91) include individuals whose oncogenicity has not yet been fully defined [4,5]. There are World Health Organization (WHO) strategies for cervical cancer prevention and screening, yet the annual cervical cancer incidence and mortality rates still force the global community to explore ways to improve current approaches for its prevention and early detection [1].

In 2018, according to WHO, the number of new cervical cancer cases was 569,847 (third most common among oncological diseases), and the number of deaths from the disease was 311,365 (accounting for one-third of all cancer deaths in females), exceeding colorectal cancer deaths in females and males combined (310,394). In 2020, at the peak of the COVID-19 pandemic, these numbers were 604,127 (third) and 341,831 (third), respectively [6,7]. However, the incidence varies widely, ranging from 4.1 per 100,000 women in West Asia to 40.1 per 100,000 women in East Africa, with mortality rates varying from 1.6 per 100,000 women in Australia and New Zealand to 28.6 per 100,000 women in East Africa [7,8,9]. 

Several problems hinder a considerable and steady reduction in new cases and overall mortality from cervical cancer. These are limited opportunities for primary prevention in low- and middle-income countries (in June 2020, only 107 [55%] WHO member countries were vaccinated against HPV, while the global estimated immunisation rate was about 15% of the adequate level), and there is difficulty in providing early screening for precancerous cervical lesions [9,10,11]. Nowadays, the most common ways to ensure the timely and effective treatment of precancerous cervix lesions include studying targeted treatment strategies, identifying early molecular markers and determining the effect of vaginal microbiota on virus clearance [12,13,14]. One of the least studied areas for improving screening programs is the application of artificial intelligence to ensure high-quality screening in regions lacking qualified specialists [15].

This study explores major problems of cervical cancer prevention strategies in several regions with varying incidence and mortality rates. 

## 2. Materials and Methods

An analysis of data published in the National Library of Medicine (Pubmed database) over the past five years has made it possible to assess the effectiveness of various approaches proposed by the national healthcare systems of the study regions. The search was conducted using the following keywords: “cervical cancer prevention”, “cervical cancer screening”, “barriers to cervical cancer prevention”, “premalignant cervical lesions” and “current strategies”. At the final stage, we searched and analysed publications focusing on new approaches to cervical cancer screening and prevention, which can further increase the effectiveness of the existing WHO strategy and national healthcare systems.

## 3. Current Cervical Cancer Prevention Strategies

In order to reduce the global cervical cancer incidence rate and decrease the social significance of this problem, in 2020, WHO adopted a global strategy to accelerate the elimination of cervical cancer. The strategy takes into account the existing differences in the capabilities of national healthcare systems [16]. The strategy sets the following target indicators (the 90-70-90 targets): 90% of girls vaccinated against HPV by the age of 15; 70% of women having undergone primary screening by the age of 35 and, for the second time, 45; 90% of women with precancerous cervical lesions and 90% of women with invasive cancer timely treated. The World Health Organization and other guidelines recommend HPV testing, including the Pap test, which is now considered a secondary procedure [1]. In the event of a positive or abnormal result, the HPV-DNA test procedure will involve colposcopy and a related biopsy [1,5]. Conversely, if HPV tests and cytological examination results are discordant, repeating the HPV-DNA test one year later and at 12–24 months (in the case of negative results or colposcopy in women who have tested positive) is recommended. Finally, in cases of CIN2 histological diagnoses, patients should be offered a surgical solution followed by a targeted follow-up over time [1].

According to the numerical model provided by the strategy developers, the implementation of the strategy can decrease the median incidence rate by 42% by 2045 and 97% by 2120 [16]. 

As the full worldwide implementation of this global strategy is difficult to achieve, it is reasonable to consider current national strategies for cervical cancer prevention and early detection.

### 3.1. Countries with Low Incidence and Mortality Rates

**Figure 1:**
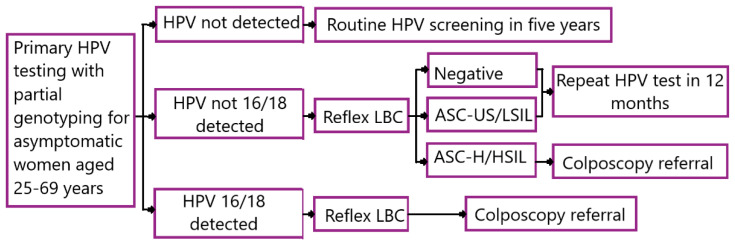
The Australian model of early screening of precancerous cervical lesions (Reprinted from Ref. [15] (do not require permission)). LBC stands for liquid-based cytology; ASC-UC, atypical squamous cells of undetermined significance; ASC-H, atypical squamous cells cannot exclude high-grade squamous intraepithelial lesions; LSIL, low-grade squamous intraepithelial lesion; HSIL, high-grade squamous intraepithelial lesion.

Australia is a representative example of an effective national cervical cancer prevention strategy with cervical cancer incidence and mortality rates at 5.6 and 1.6 cases per 100,000, respectively, as estimated in GLOBOCAN [7]. The key feature of its HPV immunisation programme is vaccinating both girls and boys with tetravalent and nonavalent vaccines [17,18]. Australian highly standardised cervical pre-cancer screening programme (Figure 1) differs from the strategy developed by WHO [1,19].

Despite the national strategy’s proven effectiveness, the country actively seeks ways to improve it within the ‘Pathways-Cervix’ project [19]. Some of the proposed revisions are listed below.

### 3.2. Countries with High Incidence and Mortality Rates

Eastern and Southern Africa both have the highest cervical cancer incidence and mortality rates. Thus, the effect of implementing the WHO strategy should be most profound in its countries [1,7,16]. We provide an analysis of the available data on the region’s current cervical cancer prevention strategies below.

According to GLOBOCAN 2020, the incidence and mortality rates in the Southern African Region were 36.4 and 20.6 per 100,000 women, respectively [7]. At the same time, Cari van Schalkwyk et al. from South Africa stated that the activities carried out in the country within the framework of the WHO global strategy prevented approximately 8600 cases of cervical cancer over the past 20 years [20]. They emphasise that WHO’s strategies focus on reducing incidence and mortality rates in the long term (targets set for 2045 and 2120). At the same time, given the current incidence rate, there is a need to search for ways to enhance existing prevention strategies affecting epidemiological indicators in the coming years and decades [20,21]. According to van Schalkwyk et al., the following issues are the major barriers to achieving acceptable incidence and mortality rates in the near future:

- The use of bivalent vaccines against HPV rather than polyvalent ones [20,21];
- Lower vaccine effectiveness in HIV-infected females [20,22,23];
- Persistent HPV infection in at least 15% of patients treated for precancerous cervical lesions [20,24];
- The use of LBC rather than HPV-DNA testing as a primary screening method [20,25].

According to estimates, amendments to the national cervical cancer prevention strategy in South Africa made in response to the above issues can contribute to the achievement of WHO-recommended levels in 10–12 years [20]. 

As discussed, the countries of East Africa show the highest cervical cancer incidence and mortality rates [7]. Healthcare systems and prevention strategies differ across countries in this region. However, individual publications provide a general understanding of how the WHO global strategy is implemented there [26,27,28]. For example, Corrado et al. from Uganda report that visual inspection with acetic acid (VIA) has an extremely low positive predictive value (PPV) of about 16%. as a method of primary screening for precancerous cervical lesions. At the same time, they note that its effectiveness does not depend much on the experience of the specialist or the location of the test (at a specialised centre or “at home”) [26]. Mchome et al. from Tanzania provided statistical data from the CONCEPT study, without referring explicitly to a national cervical cancer prevention strategy or describing HPV vaccination coverage [27]. In the country, 18.9% of women have HPV [27]. Describing an attempt at screening for precancerous cervical lesions in northwestern Ethiopia, Destaw et al. cited disappointing statistics: out of 493 women selected for the study, only 76 (16.4%) were screened (VIA) [28]. The authors do not provide data on the existence of a national strategy for cervical cancer prevention, indicating insufficient awareness of the disease and a low literacy rate as the main factors impeding adequate screening [28].

### 3.3. Countries with Intermediate Incidence and Mortality Rates

Jingfen Zhu et al. from China pointed out that despite China’s rather low cervical cancer incidence and mortality rates (10.2 and 5.3 per 100,000, respectively) compared to the above regions, the prevention and early detection of precancerous lesions in this country are acute problems [7,29]. China accounts for 11.9% of the global cervical cancer mortality rate, with the number of new cases per day reaching 12.3% of the global number in 2017 [30,31]. Jingfen Zhu names the high cost of vaccines as the main obstacle to mass HPV vaccination in the country [24]. In 2009, to achieve the cervical pre-cancer screening targets, China initiated the National Cervical Cancer Screening Program in Rural Areas (NACCSPRA). It involves DNA-HPV testing in some provinces, as well as extensive cytology and colposcopy coverage [29]. By 2019, the measures allowed engaging approximately 120 million women in the screening (21.4% of the required number). However, recent mathematical models reflect a continuing trend towards a growth in the incidence rate in the coming years. This could be due to an increase in the early detection of cervical cancer and quite a low uptake of preventive measures in females [30,32]. According to Jingfen Zhu et al., the main reason for the low effectiveness of screening in China is insufficient funding for the NACCSPRA programme, hindering the development of information systems, ways of promoting public awareness and the expansion of public engagement [30].

Indirect data show that, in 2020, the cervical cancer incidence and mortality rate in Russia were 14.1 and 6.1 cases per 100,000, respectively (the International Agency for Research on Cancer, November 2022) [33,34]. According to Tatarinova et al., the cervical cancer detection rate during active screening does not exceed 40%. It is impossible to keep track of participation in primary prevention programmes despite the presence of a national strategy for early screening of precancerous cervical diseases [34,35]. The main problems impeding the widespread implementation of the WHO strategy in Russia are as follows [36]:

- No HPV vaccination in the national immunisation schedule;
- No domestic HPV vaccine is allowed for use;
- No binding national standards on the early screening of precancerous cervical lesions.

### 3.4. Problems Common to Current National Strategies

It is generally accepted that state revenues determine a country’s ability to follow WHO’s global strategy to accelerate the elimination of cervical cancer [1,7,16], yet some problems are common to all countries.

According to recent reports by the WHO and the United Nations Children’s Fund (UNICEF), in 2019, less than a third of girls lived in countries with mandatory HPV vaccinations on national schedules. Moreover, even among them, many were not immunised regardless of the state’s revenue level [37,38]. Jacqueline Spayne et al. estimated that, in 2018, 15-year-old girls vaccinated against HPV accounted for only 12.5% of the global cohort (61 million); up to 7000 of them, almost all from low-income countries (LICs), run a high risk of dying from cervical cancer [38]. 

One of the major problems in primary prevention, inherent in not only low-income but also middle- (MICs) and high-income (HICs) countries, is the low awareness among both women and men of the disease and its prevention [39,40,41]. For instance, a survey of young sexually active men in the United States conducted by Jennifer A. Sledge et al. shows that only 64% of them had heard of the infection caused by HPV, while 86% did not know of the vaccines against HPV for women [42]. A survey among Korean adult men, conducted by Hae Won Kim et al., indicated that the majority of respondents, half of them married, were not familiar with the ways to effectively prevent the disease or understand its social significance [40]. Raising the population’s awareness of cervical cancer prevention and early detection requires not only scaling the use of modern digital technologies but also the active engagement of men [41,42,43,44].

Another equally important issue is switching from the screen-and-treat principle to the screen, triage and treat principle when managing patients with cervical abnormalities, including pre-cancer abnormalities [1,45,46]. Van Nghiem et al. from the USA argued that the “screen-and-treat” (see-and-treat) strategy makes it possible to reduce the number of women who drop out when colposcopy is required on the one hand; on the other hand, it entails excessive treatment and may be economically unviable in some cases [46]. Despite the fact that it significantly increases the burden on primary care and the number of invasive procedures, this approach to the treatment of precancerous cervical lesions has become common in LICs as they lack funds for high-quality triage [47,48]. Considering different situations across regions, the wider adoption of the “screen, triage and treat” strategy requires its modification with a view toward optimizing follow-up schedules and establishing clear indications for the mandatory invasive treatment of cervical diseases [1,19,48,49].

## 4. Prospects for Improving Primary Prevention

Table 1 shows the main challenges faced by countries that try to achieve the goals of the WHO strategy for cervical cancer prevention. 

The data analysis indicates that the common causes of failure to implement the principles of the strategy are the need for large-scale public investments for preventive healthcare, the heavy burden on primary care and the lack of HPV vaccines in MICs and LICs. One of the most affordable ways to implement the primary prevention of cervical cancer is increasing public awareness of its contribution to female cancer deaths and the potential reversibility of precancerous changes in the cervix, yet there is room for development in all countries regardless of their revenues. 

Although the targets of the WHO strategy are still far from being achieved, some countries continue to search for other ways to improve primary cervical cancer prevention. The literature review shows that most authors found the following measures promising beyond achieving the “90-70-90” targets:

- Studying the effectiveness of HPV vaccination in women over 35 years of age [70,71,72];
- Increasing vaccination uptake in men [19,73,74];
- Developing new HPV vaccines to expand preventive vaccination in MICs and LICs [75,76]

## 5. Prospects for Improving Secondary Prevention (Screening)

Although primary cervical cancer prevention can have a significant impact on the incidence rate in the long term, it does not reduce the incidence rate in women who are at risk right now. Even representatives of countries with the lowest cervical cancer incidence and mortality rates recognise the need to improve the “screen, triage and treat” strategy [19]. Table 2 shows the promising areas of secondary prevention according to Velentzis et al. from Australia.

Several more actively studied ways to improve the “screen, triage, and treat” approach will be considered below.

### 5.1. Acceptability of Self-Sampling for Pre-Cancer Screening: Screen and Triage

Although the first reports on developing methods for self-collection for cervical screening date back to 1982, there were no further reports over the next 18 years on its effectiveness or implementation in healthcare [77,78]. For instance, in 1982, Noguchi wrote that the Kato technique for the cervical smear collection with a subsequent cytological study had similar results to the classical method of obtaining materials. However, no convincing statistical evidence was provided [77]. The next reports on the possibilities of vaginal smear self-collection, now for HPV testing, came at the beginning of the 21st century [78,79]. For example, in 2000, Sellors studied the collection of vulvar, vaginal and urethral samples for HPV detection by using a hybrid capture II assay (Digene Corp., Silver Spring, Md.) [78]. The results show that the sensitivity for self-collected samples ranged from 44.8% to 86.2%, and the specificity ranged from 53.5% to 69.7%. For the samples collected by physicians, the sensitivity was 98.3%, and the specificity was 52.1% [78]. In 2001, Gravitt et al. reported a high consistency of PCR results for HPV detection in cervical cancer patients when comparing self-collected and clinician-administered samples [79], yet at the end of the first decade of the twenty-first century, self-sampling was not a common method. Less than ten years ago, the global community again directed attention to the possibility of expanding cervical cancer screening via the self-collection of samples. Lazcano-Ponce et al. presented data on successful HPV screening in more than 100 thousand women using self-collected samples. However, the authors did not compare the effectiveness of this technique and the classical method [80]. Duke et al. from Canada calculated that self-sampling caused a fivefold increase in the intensity of screening compared to the classical approaches. However, the authors did not provide data on either the method’s sensitivity or specificity [81]. Comparing two vaginal self-collection methods and clinician-assisted sampling, Haguenoer et al. demonstrated a high efficiency of self-sampling and a high correspondence of its results to those obtained from the classical sampling (from 90 to 97%) [82]. The beginning of the COVID-19 pandemic coincided with an increase in the number of publications on self-sampling effectiveness. In addition, WHO officially recommended self-sampling as part of the cervical cancer prevention strategy [1,16]. Over the past three years, at least 80 papers have been published on this topic. There are ongoing investigations comparing the results of HPV testing of samples obtained via self-collection with the results of VIA, as well as population-based studies on the possibilities of implementing this technique in LICs and MICs or using it among the socially disadvantaged groups in HICs [83,84,85].

Currently, the most active research area is the detection of not only HPV DNA copies but also markers of malignancy in self-collected samples. For instance, back in 2014, Verhoef et al. concluded that it was possible to use DNA methylation-based triage on self-sampled specimens [86]. The study, having high statistical power, showed the similar effectiveness of molecular and cytology triage before referral for colposcopy [86]. Hesselink presented similar data showing the MAL-m1/miR-124-2 sensitivity for the detection of CIN3+ of up to 87.8% [87]. In 2022, authors from China presented data on the triage performance and predictive value of human gene methylation panels (ZNF671/ASTN1/ITGA4/RXFP3/SOX17/DLX1), including a combination of HPV16/18 genotyping, studied on self-collected samples [88]. To date, WHO does not regulate the use of such technologies for the early screening and prevention of cervical cancer, but further research in this direction looks promising.

### 5.2. Digital Technologies for Detection of Precancerous Cervical Lesions: Screen and Triage

Today, the use of digital technologies in cervical cancer screening can ease the burden on primary care. In 2018, Ravikumar et al. reported on enhancing Clinical Decision Support Systems (CDSS) intended to assist primary care clinicians in making decisions based on electronic medical records [52]. The introduction of this system to determine the tactics of patient treatment after cervical cancer screening made it possible to improve the care recommendation accuracy up to 94% [52]. 

Using artificial intelligence to interpret primary screening results is of particular interest for screening programme enhancement. In 2020, Elima Hussain et al. reported the completion of a training dataset for the automated assessment of Pap test results but did not submit a finished product [89]. In 2021, Li X et al. from China presented a neural network capable of replacing medical laboratory scientists in interpreting liquid-based cytology results in the future, potentially solving the problem of personnel shortages in MICs and LICs [90]. In the same year, Anabik Pal et al. from the USA presented a similar development with a diagnostic accuracy of 84.55% [91]. Pirovano et al. presented the results of machine learning on liquid-based cytology samples. The accuracy of abnormal smears detection was 95.2%, with only 66.8% accuracy on severity classification (LSIL and HSIL) [92]. As part of a study on the effectiveness of their automatic model, Xiangyu Tan et al. conducted a retrospective analysis of the accuracy of the system using the training dataset of 13,775 ThinPrep cytological test (TCT) images. Its sensitivity for ASCUS, LSIL and HSIL was 89.3%, 71.5%, and 73.9%, respectively, while the specificity of the proposed system in determining smear abnormalities was only 34.8% [15].

Another application of artificial intelligence in cervical pre-cancer detection is digital colposcopy using artificial intelligence to facilitate the work of a primary care physician. Liming Hu et al. in 2019 showed that their “deep learning”-based algorithm ensured a fairly accurate detection of CIN2^+^ by analysing cervical images. However, the authors were cautious in interpreting the study’s results [93]. Studying the possibility of using a machine learning algorithm for smartphone-based visual inspection after acetic acid (VIA), Bae et al. approached the possible introduction of digital technologies in limited-resource settings. However, the small size of the studied group does not allow us to draw conclusions on the potential of the proposed tool [94]. Xue et al. also suggested using artificial intelligence to improve the accuracy of cervical biopsy during colposcopy [95]. AI-guided colposcopy can be implemented in regions lacking highly qualified specialists [95]. Another approach increasingly employed to improve the quality of diagnostics using digital technologies is a cross-modal integration of data obtained during colposcopy, cytology and HPV testing [96,97]. 

### 5.3. Studying Vaginal Microbiome as an Integral Part of HPV Pathogenesis: Screen and Triage

The term “microbiome” was coined by Lederberg and McCray in 2001 to define a set of all microorganisms sharing one living space and interacting with the surrounding host tissues (human organism) [98]. For a long period of time, the only pathological conditions associated with a change in the composition of the normal vaginal microbiota (except for sexually transmitted infections) were bacterial vaginosis (*Gardnerella vaginalis*) and vaginal candidiasis [99,100]. Later, studies have shown that the state of the vaginal microbiome is a dynamic indicator influenced by many factors, such as sexual activity, hormonal disorders, hygienic habits, lactation, stress, diabetes, diet, region of residence and ethnicity [101,102,103]. In 2011, Ravel et al. performed the pyrosequencing of barcoded 16S rRNA genes of the vaginal samples of 396 women to cluster the “normal” female microbiome into 5 groups [104]. They presented their findings in the form of a heat map of log10-transformed proportions of microbial taxa found in vaginal bacterial communities (Figure 1 in the original article) [104]. The first group included the types of microbiomes in which *L. crispatus* prevailed; the second type included *L. gasseri*; the third included *L. inners*. The fourth type of microbiomes was characterised by the predominance of *L. jensenii*. All other microbiomes were assigned by the authors to the fifth type [104]. This approach made it possible to change the perception of the “normal” vaginal microflora. The fundamental mechanisms of bacterial microbiome’s effect on the processes in the cervix epithelium in the case of an HPV infection have not been studied sufficiently. According to the latest data, *L. iners* is not capable of producing D-lactic acid and reactive oxygen species, albeit Lactobacillus species usually produce lactic acid, H_2_O_2_ and antimicrobial peptides such as bacteriocins and biosurfactants that inhibit the growth of bacteria and viruses and regulate vaginal homeostasis [101,105]. According to the results of the studies by Petrova et al. and Rampersaud et al., L. iners produces L-lactic acid and inerolysin (a pore-forming toxin cytolysin capable of damaging the cells of the vaginal mucosa), both contributing to pathogenic proliferation and infections [106,107]. Major research conducted in recent years confirms the role of microbiome in virus clearance and oncogenesis [108,109].

In 2017, Di Paola et al. found that *Gardnerella*, *Prevotella*, *Megasphoera* and *Atopobium* were present at sampling in 43% of women in the persistence group and only in 7% in the clearance group when examining the microbiome from women with high-risk HPV persistence [110]. They assumed that *Atopobium* spp and sialidase gene from *Gardnerella vaginalis* were independent markers of HPV persistence [110]. In 2020, based on a meta-analysis, Norenhag et al. [111] linked microbiota dominated by non-lactobacillus flora or L. Iners to a three to five times higher risk of dysplasia/cervical cancer for any prevalent HPV and two to three times higher risk of high-risk HPV, compared with *L. crispatus*. In 2021, Kang GU et al. reported that the microbiota composition in invasive cervical cancer and CIN is significantly different from the microbiome of healthy women. The authors suggested that *Gardnerella* and *Streptococcus* may be involved in carcinogenesis [112]. In 2020, Mitra et al., in a study of the effect of vaginal microbiome on spontaneous CIN2 regression and HPV clearance, obtained data suggesting that the predominance of non-lactobacillus flora (*Prevotella*, *Atopobium* and *Gardnerella*) was associated with virus persistence, and the predominance of *L. crispatus* and *L. gasseri* was associated with more frequent regression and clearance [14]. In the same year, Mei Yang et al. found that Trichomonas vaginalis and HPV16 co-infection were associated with a 1.71-fold increase in CIN 2-3 risk [113]. The results of a study published at the end of 2022 are interesting as well: Miriam Dellino et al. suggested that the long-term administration of oral *Lactobacillus crispatus* can restore eubiosis in women with HPV infections and hence achieve viral clearance. Total HPV clearance was shown in 9.3% of patients undergoing follow-up only, compared to 15.3% of patients in the group taking long-term (median 12 months (range 9–14 months)) oral *Lactobacillus crispatus* M247 (*p* = 0.34) [114]. Despite the percentage of HPV-negative patients assessed with the HPV-DNA test at the end of the study period (being similar to the control group), further research studies in this direction seem promising.

A further study of the effect of vaginal microbiome on HPV clearance and persistence, as well as carcinogenesis, opens up new opportunities for both molecular-based triage and the treatment of patients with HPV or abnormal changes in the cervical epithelium.

## 6. Conclusions

At the moment, the ability of countries to achieve the targets of the WHO global strategy to accelerate the elimination of cervical cancer is limited by the level of their socioeconomic wellbeing. 

To increase the effectiveness of primary prevention measures, researchers from different countries propose the following:

- Raising awareness of HPV risks and the virus’s role in oncogenesis among not only females but also males;
- Inclusion of HPV vaccination of boys in national immunisation schedules;
- Development of new HPV vaccines to expand vaccination to MICs and LICs.

To increase the effectiveness of secondary prevention measures (early screening for precancerous cervical lesions), the following is proposed.

- A more intensive study into the effectiveness of the analysis of self-collected samples;
- Search for molecular markers of carcinogenesis in self-collected samples;
- Using artificial intelligence to detect abnormalities in samples for PAP testing;
- Using artificial intelligence in guided cervical biopsies to improve the accuracy of histology;
- Development of diagnostic and therapeutic approaches for treatments based on vaginal microbiome analysis.

## Figures and Tables

**Table 1:** Problems facing countries with different revenue levels in implementing the Global Strategy to Accelerate the Elimination of Cervical Cancer as a Public Health Problem.

State	Group of Countries by Revenue Level	Authors	Main Problems
Australia	HICs	Lew et al. [17] Velentzis et al. [19]	Difficulties in involving indigenous people in screening programmes. Difficulty in spreading screening programmes to socially disadvantaged groups.
USA	HICs	Baezconde-Garbanati et al. [50] Castle et al. [51] Ravikumar et al. [52] McGinnis et al. [53]	Insufficient screening uptake among immigrants in the southern states. Low awareness of cervical cancer screening and prevention among women. Frequency of screening often exceeds three years. Heavy burden on primary care and difficulties in interpreting borderline screening results.
Republic ofKorea	HICs	Kim et al. [40] Shin et al. [54] Min-A Kim et al. [55] Jaehyun Seong et al. [56]	HPV vaccination coverage below 5%. Early sexual activity. Low awareness of the role of HPV in the development of cervical and other cancers. Use of only Pap testing in screening precancerous cervical lesions.
China	MICs	Zhu et al. [29] Xia et al. [30] Wang et al. [57] Yuan-Yuan Zhang et al. [58]	Low screening uptake in rural areas. Insufficient public awareness of cervical cancer. Insufficient funding for government screening programmes. No HPV vaccine developed and manufactured in China. Insufficient spread of the “screen, triage and treat” approach.
South African Republic	MICs	Van Schalkwyk et al. [20] Jennifer Ducray et al. [59]	Unavailability of quadrivalent and nonavalent vaccines in the region. Lower vaccine effectiveness in HIV-infected females. Insufficient PCR testing. Low public awareness of cervical cancer prevention and screening. Lack of engagement of the poorest strata in prevention activities.
India	MICs	Neerja Bhatla et al. [60] Joshi et al. [61] Neha Taneja et al. [62]	Uneven vaccination coverage of the country’s states. Low awareness among women of the disease’s causes, development and prevention. High rate of treatment refusal after a positive primary screening test. Low adherence to treatment and follow-up in women using mobile screening centres.
Uganda	LICs	Corrado et al. [26] Isabirye et al. [63] Carolyn Nakisige et al. [64]	A high rate of false positive VIA test results. Limited availability of HPV vaccines. Low awareness of the development and prevention of the disease among women. No standardised state screening and prevention programme.
Malawi	LICs	Cubie et al. [65] Kakani et al. [66] Gerstl et al. [67]	Impossibility of public funding for programmes within WHO’s strategy. No state HPV vaccination programme. Low motivation of women to participate in the active screening programme. VIA testing as the only screening method. Unavailability of primary HPV-DNA testing.
Liberia	LICs	Afzal et al. [68] Beddoe et al. [69]	Healthcare infrastructure destroyed in the long civil war. Religious restrictions on active screening of females. Low level of public awareness of cervical cancer and its prevention. Self-collection as the predominant sampling method.

**Table 2:** Promising ways to enhance secondary cervical cancer prevention according to Australia’s Scientific Advisory Committee (SAC) (Adapted from Ref. [15] (do not require permission)).

Evaluation	Approach
Optimal screening regime for unvaccinated women (based on birth cohort) and vaccinated women based on their vaccination history and type of vaccine received.	Tailored screening based on vaccination status (HPV4 or HPV9).
Longer interval screening schedules following two consecutive negative HPV test results within routine screening.	Tailored screening based on vaccination and screening history.
Impact of partial genotyping for oncogenic HPV types other than 16/18 with direct colposcopy referral for select types compared to cytology triage.	Methods for triage.
Impact of triaging oncogenic HPV-positive (non 16/18 types) women with dual-staining (p16 ki67) cytology compared to LBC.	Methods for triage.
Impact of methylation markers in HPV-positive self-collected samples testing compared to clinician-collected cytology test.	Methods for triage.
Impact of vaccinating women treated for CIN2/3 with HPV4/HPV9 if the vaccine reduces recurrence by 50% (for pre-existing HPV types) or 80% (naïve for HPV types).	The vaccine to prevent CIN2/3 recurrence.

## Data Availability

Data sharing not applicable. No new data were created or analysed in this study. Data sharing is not applicable to this article.
